# Political analysis in public health: middle-range concepts to make sense of the politics of health

**DOI:** 10.1093/eurpub/cky159

**Published:** 2018-11-01

**Authors:** Scott L Greer, Marleen P M Bekker, Natasha Azzopardi-Muscat, Martin McKee

**Affiliations:** 1 University of Michigan School of Public Health, Ann Arbor, MI, USA; 2 Department of Social Sciences, Chair Group Health and Society, Wageningen University and Research, Wageningen, The Netherlands; 3 Department of Health Services Management, University of Malta, Msida, Malta; 4 London School of Hygiene and Tropical Medicine, London, UK

## Abstract

Public health is about policy, power, and the public and as such might be thought necessarily political. That does not mean, however, that the place of political analysis and engagement in public health is uncontroversial, and there have been longstanding arguments that to discuss politics sullies the scientific nature of public health. This article, introducing a special issue on political science in public health, argues that rigorous use of middle-range theory can inform our analysis of public health problems and avoid the risks of politicization, excessive abstraction or excessive concreteness. It summarizes key political science concepts discussed in the papers: epistemic communities, interest groups, advocacy coalitions, political parties, institutions, legalism, discourse and the political economy of labour. We hope that the series will provide the public health community with some tools and methods for how to integrate public health knowledge into the sphere of decision making in an appropriate way.

## Politics matters

Just over two decades ago an editorial in The Lancet^1^ kicked off a debate in the public health community that remains unresolved today. It argued that public health was losing its way, with epidemiologists focusing ever more narrowly on molecular and genetic causes of disease in individuals while ignoring the upstream factors that determined the burden of disease in populations. However, it also laid down a challenge to those arguing for a broader perspective, pointing to the enormous difficulties of researching the larger forces involved.

Some of the leading experts in public health and epidemiology weighed into the debate. At its heart was the argument by Beaglehole and Bonita^2^ that ‘for many epidemiologists the study of social factors is considered too political… it is necessary for epidemiology to affirm its connection with policy and to reject scientific isolation’. In response, Rothman and colleagues leapt to the defence of epidemiologists, with a firm *no* to the question ‘Should the mission of epidemiology include the eradication of poverty?’,^3^ stressing the challenges of changing society and the benefits of risk factor epidemiology. Pearce, in turn, argued that ‘the decision *not* (emphasis in original) to consider population factors is itself a political one’.^4^

Today, the argument has moved on considerably. Then, Rothman and colleagues argued that ‘Many applications can be carried out directly by individuals … information about the risks of having unprotected sex can persuade people to change their sexual behaviour, and information about the risks of smoking can motivate smokers to give up their habit’. Now it is widely accepted that information is rarely adequate to bring about behavior change. Much more is also known about patterns of risk factors and disease, and the factors that influence both. The concept of social determinants of health^5^ is now established and has, more recently, been joined by political^6^ and commercial determinants.^7^ Thus, beside the expected papers on, for example, the role of salt in cardiovascular disease,^8^ the Lancet has recently published papers on the health effects of the UK’s decision to leave the European Union^9^ and of the Trump administration.^10^

Of course, in some ways, none of this is new. In his 1848 report on the epidemic of typhus in Silesia the pathologist Rudolf Virchow bemoaned how the people, oppressed by the aristocracy, ‘silently died of starvation’.^11^ His remedy was unapologetically political, ‘full and unlimited democracy’, and a list of things that had to be done to prevent such a tragedy happening again, including fairer taxes and workers’ rights. What he did not do was say how these things might be brought about. Here, Rothman and colleagues may have a point. Even when it knows what should be done, and they cite as an example the abolition of nuclear weapons, the public health community is unable to do anything about it. Yet there have also been many examples of where public health advocacy has brought about change, such as bans on smoking in public places, higher taxes on alcohol, and road safety measures.^12^ Sometimes they succeed but sometimes they fail. And the reasons are often political. So how can public health professionals understand the political dimension to their work? This is the subject of this series of articles.

## Key tools for political analysis in public health

There are several obvious risks in trying to incorporate the analysis of politics into public health research and action. The first concerns preserving scholarly autonomy when thinking about politics. On one hand, work can be diminished or denigrated if identified with a political party or interest. On the other hand, and equally problematic, is the denial of the impact of politics, or a nihilistic insistence that ‘they’re all the same.’ They aren’t.^13^ What happens if research finds that a particular political force is directly bad for health, as can easily happen when we look at the impact of policies favoured by one party or opposed by another? It is difficult to avoid appearing partisan in such circumstances. Indeed, one can be accused of partisanship even if one’s professional self-presentation is one of arid scholasticism. The accusation of partisanship often depends on the politics of the accuser. Moreover, what is seen as partisan changes. Things that would once have been taken for granted, such as slavery or denial of female suffrage, are no longer so. Once, anyone opposing them could easily have been accused of political partisanship. It is possible, using appropriate framing and discourse, to influence such norms, by shifting what is termed the Overton Window.^14^ The evidence produced by public health researchers can play a role in this process, with recognition of this fact likely explaining the ban on using Federal funding in the USA to research firearms injuries,^15^ lest the scale of the problem and the potential remedies enter popular discourse.

A second kind of risk is about keeping an appropriate perspective on the political system. It is tempting to slide into macrosociology when thinking about public health, using very big concepts like capitalism, populism and neoliberalism which can get in the way of precise empirical analysis. A common alternative is to shift to a very micro level, attributing success and failure to the political will of an individual politician or the communicative skills of a few activists or forgetting elected politicians and focusing on bureaucrats with specialist expertise. Both offer valuable insights but neither, on its own, is satisfactory. One leaves us with giant concepts such as capitalism or structural violence whose specific content cannot be seized, and one leaves us with findings that might not generalize to the other side of town, much less other countries.

The solution to both problems, of maintaining impartial and rigorous scientific standards while studying politics, is to focus political inquiry on theories of the middle range. Theories of the middle range are: ‘intermediate to general theories of social systems which are too remote from particular classes of social behavior, organization, and change to account for what is observed and to those detailed orderly descriptions of particulars that are not generalized at all. Middle-range theory involves abstractions, of course, but they are close enough to observed data to be incorporated in propositions that permit empirical testing. Middle-range theories deal with delimited aspects of social phenomena, as is indicated by their labels’.^16^

Middle range theories allow us to be confident in our science and the scope, conditions, and applicability of our findings. They allow us to look at trees and forest in delimited slices, with a focus on what we are looking at. They are the ones which get the most traction in political science and form the core of its contribution to the analysis of politics and policies.

The purpose of this special issue is to illustrate some key middle range theories of politics and how they are useful in explaining policy in some key areas for public health: vaccines, obesity, pharmaceutical policy, income equality and tobacco control. Our approach was to commission articles that would each combine a powerful conceptual tool for political analysis with a topic of compelling public health interest.

## Epistemic communities and health technology assessment

One of the most basic problems is how scientific public health findings can be brought to affect policy. Olga Löblová’s article addresses this.^17^ She criticizes the standard models that posit a direct line from public health research to scholarly impact:^18^ ‘Experts often see themselves as heroes in an epic: they must swim seven seas (“bridge gaps between science and policy”), climb seven mountains (“overcome barriers to adoption of research”), and translate their wisdom into a foreign tongue for the king to understand (“engage in knowledge translation”). When they fail, their default response is to try harder: set up more knowledge transfer initiatives, employ more science communication professionals.’

The concept of an *epistemic community* allows us to escape this heroic narrative, which as often as not ends in frustration for the would-be hero, and better understand public health’s own place in politics and policy. An epistemic community is a network of professionals united by a shared set of normative beliefs, shared causal beliefs about how the world works, shared notions of validity and evidence, and a common policy enterprise. Public health is one such epistemic community. Löblová’s example is Health Technology Assessment, an epistemic community that gained influence at different times in different parts of Europe but which has had an unexpectedly difficult time influencing power at the member state and EU level. By tracing the ways epistemic communities interact with other political forces, she shows us how we can locate our own efforts to convince and communicate in broader political fields.

## Interest groups and pharmaceuticals policy

‘Special interest’ is an insult in common language, and to call somebody or something a ‘lobby’ still worse. But when we try to influence politics, we are lobbying, and when we organize to do it, we are interest groups. Brooks uses the *Advocacy Coalition Framework* to map out the ways pharmaceuticals policy is made in the EU,^19^ complementing Löblová’s paper on epistemic communities with a different perspective that emphasizes the complex ways people organize across institutions and political arenas to make policy.

Making or changing policy is hard and takes many kinds of efforts by many different people in and out of interest groups, government, academia, media and other organizations who form into ‘advocacy coalitions’ pushing for or against particular policies. In her case, the contest was over direct to consumer (DTC) pharmaceutical advertising in the EU. On one side stood the European Commission, allied with an advocacy coalition in favour of liberalizing rules on DTC. An alternative coalition of NGOs, professional organizations, and others joined the battle and successfully blocked the legislation. Viewing the ways that the two coalitions contested across different venues, from specialist media to EU institutions, shows the importance and strategic and tactical complexity of working in coalitions.

## Partisanship and the impact of the populist radical right

While public health researchers often focus on the dimensions of politics that they can affect, that is not the same thing as understanding the systems in which politicians operate. Expertise and credibility in public health are not the same thing as power and strategy in politics. One of the biggest factors in how politicians shape agendas and make decisions is the *political party*. Politicians play in teams, and the teams are political parties. While political parties are often unloved, individually and collectively, they also shape political careers and power, and therefore policy. Looking at politics without looking at parties and partisanship is like trying to watch football without understanding teams.

Falkenbach and Greer^13^ briefly define political parties and party systems before turning to one of the biggest, and also theoretically and methodologically challenging problems of understanding parties: how and when do they matter? Most of the literature on this topic focuses on the impact of Social Democratic and Christian Democratic parties.^20^ It finds that they both correlate with a larger welfare state but do it differently, with different consequences for equality. This paper then uses the timely example of populist radical right parties such as the Austrian Freedom Party or Swiss People’s Party to understand what impact they can and do have in government. The experience of countries where the populist radical right parties have been in government shows that they concentrate their effects on policies focused on discouraging immigration. While they often use a ‘welfare chauvinist’ argument, calling for greater benefits for the native people as well as exclusion of immigrants and minorities, in practice they rarely expand benefits and might, at most, slow cuts. Their impact, further, is limited by coalition partners and the rule of law.

## Institutions and collaboration

*Political institutions* shape the receptiveness of governments to different policy initiatives and the sustainability of different policies. Some policies are unlikely to work in some political systems. Bekker et al.^21^ make this point, using a comparison of the different fates of collaborative public health initiatives in the UK and Netherlands. In both countries, a popular kind of initiative was adopted by governments, involving collaboration with civil society and business to affect determinants of health. In the UK, it took the form of the Responsibility Deal, which failed to achieve credibility^22^ and was short-lived while the Dutch initiative, All about Health, thrived and adapted.

In part, this just illustrates the differences between the collaborative Dutch and adversarial English political cultures. But underneath the different fates of the two programmes lie deep and lasting institutional and political factors. A Dutch propensity to collaboration and a British adversarial approach are not just artefacts of culture. A collaborative model reflects Dutch institutions, including electoral rules that ensure coalition governments.^23^ Dutch politics fosters rational adhesion to collaborative norms. Meanwhile, majoritarian British institutions, including a first past the post voting system, can create a strong government, which also benefits from being unfettered by a written constitution. It is harder for British leaders to foster collaboration because everybody knows that governments will change and cannot bind their successors. Dutch politicians, companies, and civil society actors are repeat players in an endless collaborative exercise, and act accordingly, while in Britain a powerful and relatively unconstrained government fundamentally shapes the strategic landscape for any policy initiative. If a British policy is to become entrenched, it must be grounded in strong local forces that can support it through election after election.

## Legalism and tobacco control

One of the biggest changes in the politics and institutions of many European countries over the last fifty years has been the development of legalism as a tool and feature of politics. *Legalism* is a ‘mode of governance characterized by a strong emphasis on the formulation and enforcement of legal rules over less formal policies, frequent court cases, legal expertise, and a multiplicity of formal policymaking structures’.^24^ Driven by factors ranging from Europeanization to the decline of political parties as interest representation, legalism has been increasingly important in European governance, and it shapes both the content of policy and the process by which policy is enacted.

Tobacco control politics and policy are thoroughgoingly legalist. The policy tools are regulatory, filtered through an analysis of typically domestic, European, and international legal challenges. The policymaking process, likewise, is conducted with scrupulous care in anticipation of legal challenges. That care and worry is justified, since the tobacco industry mounts creative and well-funded legal challenges to policies it opposes.^25^ Advocates of tobacco control, inspired by activists in the United States, also try to use legalism to their advantage, litigating against the industry. The political terrain of tobacco control is very much the law, and understanding legalism is as necessary as understanding science, lobbying, or political parties in understanding the politics of tobacco control today.

## Discourse, the state, and vaccines

Even if policymakers are persuaded, even if the policy is enacted, it can still face resistance in implementation. Resistance is often a result of deep disjunctures between different circles of elite and popular conversation. Nowhere, perhaps, is that more evident than vaccine hesitation, the case Kieslich uses to illuminate the challenges of state-society relations.^26^ Public health, from its inception has worked with and through the state, but that means all too often that public health initiatives can share in suspicion of state power. Kieslich focuses on the concept of *discourse coalitions*, which is a set of narratives and people who promote them. Their propagation, often through the internet and social media, encourages individual resistance and reframes debates. In the case of vaccines, for example, the vaccine-hesistant discourse coalition converted the topic being discussed from technical public health issues to the legitimacy of state power. Failure to understand this discourse shift makes many aspects of vaccine politics unintelligible, notably the propensity of populist politicians to adopt anti-vaccination stances. Their stances are more views about state power than about communicable disease prevention.

## Labour markets and equality

It is a widely accepted finding in public health that more equal societies have better population health and lesser health inequalities. But understanding the implications of that finding requires a much broader understanding of comparative *political economy*.^27^

There are three major ways in which states structure people’s life chances: the welfare state (including the health system), industrial relations (wage-setting and bargaining institutions) and labour market regulation (rules on hiring and firing). Public health research, for all its concern with inequality, frequently forgets the second two. The result is much public health thinking, focused on taxing and spending, which misunderstands the range of policies that promote equality and inequality. The welfare states of the United Kingdom and Sweden are roughly equally redistributive. It is the pre-tax, pre-benefit egalitarianism of Sweden that makes the difference, and that is caused by their different labour regulation and industrial relations systems. Danish ‘flexicurity’ likewise depends on the highly organized Danish labour market as much as on Danish taxes and welfare programmes.

The article discusses the different ways in which industrial relations and labour regulation shape health and equality, from more egalitarian wage-setting to reduced ‘presenteeism’ in which a sick employee comes to work, is unproductive, and infects others. Over and over again, the benefits of strong and coordinated unions appear in the literature, but so does evidence of union decline and the erosion of coordination in labour markets.

The article should be a corrective to accounts of health and income equality that focus only on the welfare state or on oversize concepts like ‘neoliberalism’. If we start to regard labour regulation and industrial relations as public health topics, then a whole new world of allies and public health policy tools become available. That should be cheering.

## Conclusion: middle range theory for political analysis in public health

Public health is an intrinsically interdisciplinary enterprise, bringing together the expertise, methods, and perspectives of fields from epidemiology to management and from biostatistics to health education. But it is notably weak in the analysis of the political system, for all that politics cause and can ameliorate many of the most pressing public health challenges we see today. Too many public health studies of politics are so microscopic as to have unknown external validity, or so macroscopic as to make it impossible to see or effect practical change.

Rather than reinvent the wheel, we should add political science to the interdisciplinary enterprise that is public health. This Special Issue brings out several well-established middle range theories of politics, chosen for their relevance to the kinds of policy and political problems in public health. The case studies of issues, from tobacco control to direct to consumer advertising and from obesity to vaccinations, should show how these concepts could be imported into public health and help us to address some of the deficiencies in public health analysis of politics and power.

## Funding

This open access Supplement received funding under an operating grant from the European Union’s Health Programme (2014–2020).

*Conflict of interest*: none declared.

**Key points**

- Public health is necessarily, fundamentally, political because the causes of ill health are affected by politics and because the remedies almost always involve politics.
- Valuable public health findings are therefore political and will be seen as such regardless of the authors' intent.
- Scientific research and analysis is possible in the study of politics through the use of middle-range social science theory.
- Viewing political science as one of the fields in the interdisciplinary enterprise of public health will increase public health's intellectual and practical strength.
